# An open-source JavaScript clinical neurophysiology library for education and clinical research

**DOI:** 10.1016/j.cnp.2026.02.001

**Published:** 2026-02-06

**Authors:** Sampsa Lohi, Petro Julkunen, Reetta Kälviäinen, Esa Mervaala

**Affiliations:** a Department of Clinical Neurophysiology, North Karelia Central Hospital, Joensuu, Finland; b Department of Clinical Neurophysiology, Kuopio University Hospital, Kuopio, Finland; c Institute of Clinical Medicine, School of Medicine, Faculty of Health Sciences, University of Eastern Finland, Kuopio, Finland; d Department of Technical Physics, University of Eastern Finland, Kuopio, Finland; e Epilepsy Center, Kuopio University Hospital, Kuopio, Finland

**Keywords:** Clinical neurophysiology, Electroencephalography, Electromyography, Nerve conduction studies, Open source, Web application

## Abstract

**Objective:**

We present ‘Epicurrents’, an open-source JavaScript library for processing and displaying neurophysiological signal data in a web browser.

**Methods:**

The library follows a modular architecture to enable support for multiple clinical neurophysiology modalities. It supports open standards such as the European Data Format (EDF) and Digital Imaging and Communications in Medicine (DICOM), with optional Python and Open Neural Network Exchange (ONNX) integrations for scientific signal processing. The application presented in this article is platform agnostic, requires no installation, and is usable both online and offline as a progressive web application.

**Results:**

The library has been tested in real-world educational and research projects and is used by the European Academy of Neurology for hands-on EEG-education in their congresses. While JavaScript’s memory management poses limitations for processing large recordings, architectural workarounds such as shared memory buffers and asynchronous processing have resulted in improved performance.

**Conclusions:**

The application presented here is not intended nor certified for clinical diagnostics, but its accessibility and extensibility make it a promising tool for neurophysiology education and research.

**Significance:**

Epicurrents is the first modular JavaScript library for clinical neurophysiology education and illustrates how web technologies can also enhance collaborative scientific research in the field of clinical neurophysiology.

## Highlights

• Epicurrents is the first multi-modal web app for clinical neurophysiology education.
• It can be used to read EEG files in several open file formats.
• As free and open-source software, Epicurrents is available for anyone to use.

## 1 Introduction

Most currently used software tools in clinical neurophysiology rely on desktop applications that have not been designed for widespread educational and scientific use. Installing and updating desktop applications for research and educational use may be challenging from data and cybersecurity perspectives and may not be permitted in a hospital environment, for instance, due to a lack of documented adherence to proper standards. In contrast, web applications are software that runs in a web browser. They can be completely platform agnostic and have zero footprints on the end user’s device. Web applications run in an isolated environment, meaning that they have no access to data residing on the user’s device, which improves data security compared to traditional desktop applications. Web applications can be made highly accessible and avoid the traditional issues related to distributing and performing updates on desktop software. They are ideal companions for cloud data storage, as they can be relatively simple to integrate into web-based storage and computing platforms. Although most web applications are accessed over the internet, it is also possible to design a JavaScript application that can be used offline from a single file or installed on a device as a progressive web application (PWA; Progressive web apps | MDN., 2025) for a more familiar desktop application appearance. A few web applications for reviewing clinical EEG have been developed in recent years (e.g. Persyst Mobile®, USA; Stratus EEG™ by Kvikna Medical, Iceland; Ceribell®, USA), but no comparable solution exists for most other clinical neurophysiology test modalities, including nerve conduction studies (NCSs) and electromyography (EMG).

Here, we introduce ‘Epicurrents’ (developed by SL), an open-source JavaScript library for processing and displaying neurophysiological signal data in a web browser. The application features a modular design, enabling extensions to new study modalities and file formats. As a result of this flexibility, the same library can be used to build lightweight applications with basic features for demonstrations and educational use, as well as applications with a more powerful set of features for scientific use. The resulting applications are categorised as zero footprint and platform agnostic, as they can be run on any device with a compatible web browser without the need to install any additional software. We believe our library could provide essential value for clinical neurophysiology education and research. We are unaware of any open-source solutions with a similar scope of capabilities.

## 2 Materials and methods

In this article, we outline the structure of the Epicurrents library and provide one example of its many possible applications. The Epicurrents library has been under development since 2017 by the corresponding author. The version we introduce here is the third iteration (version 0.3). Most of its source code is available on GitHub (https://github.com/epicurrents) under the Apache License, version 2.0 (https://apache.org), which allows for free non-commercial and commercial use. A test version of the software is publicly available online at https://alpha.epicurrents.io. This work did not involve human or animal subjects and hence no ethical approval was required.

**Fig. 1:**
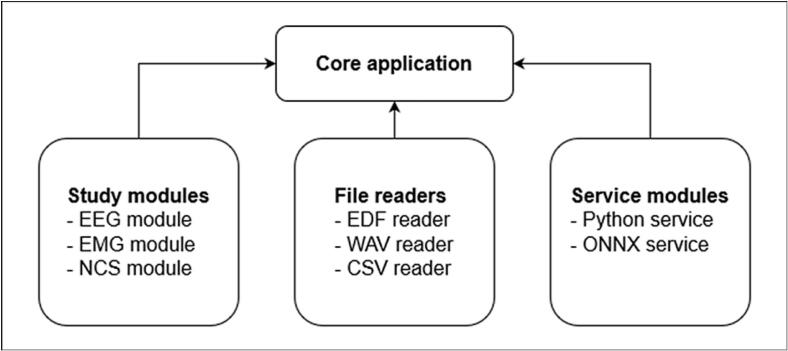
A simple schematic of Epicurrents’ library structure. Epicurrents applications require at least one study module and one file reader to be registered with the core application. Service modules are optional. Modules work independently of each other and only depend on the core application. EMG = electromyography; NCS = nerve conduction study; EDF = European data format; WAV = wave file format; CSV = comma-separated value file; ONNX = Open Neural Network Exchange.

Except for the core application library, the source code is divided into individual modules that belong to one of three categories: study modules, file readers, and service modules (Fig. 1). From the first two categories, at least one module is required to open a file and process the signal data. The modules currently under development in these two categories are listed in Table 1.

1. **Study modules**. Modules in this category add the ability to load and process data according to a specific test modality. In essence, they organise the raw signals read from a data source into meaningful neurophysiological studies. Examples of this module type include loaders for EEG, polysomnography, EMG, and nerve conduction recordings.
2. **File reader modules**. File readers can read raw data from a certain file type and format. They may be tied to a specific study type (readers for vendor-specific formats) or can be used by any applicable study loader (universal file formats). File readers should support reading data both from the user’s device and from remote data sources.
3. **Service modules**. Optional service modules add extra functionality beyond basic signal processing. Examples of service modules include a Python interpreter service that adds the capability to use Python scripts and an Open Neural Network Exchange (ONNX) service that adds the capability to use machine learning (ML) models to process the signal data.

**Table 1:** Supported file formats and manufacturers.

Modality	File types and supported manufacturers	Status
EEG/MEG	EDF/ EDF + and its extension BDF/BDF+ (universal) DICOM	Stable Functional
EMG/SFEMG	WAV (universal, requires device-specific setup) CSV (Cadwell Sierra) Study exports in text format (Synergy) As exported epochs in text format (Keypoint.net, EMG)	Stable Functional FunctionalExperimental
NCS	Study exports in text format (Synergy) Study exports in text format (Keypoint.net)	Functional Experimental
PSG	EDF, EDF+, BDF, BDF+ (universal)	Experimental

MEG: magnetoencephalography; EMG: electromyography; SFEMG: single-fibre electromyography; NCS: nerve conduction study; PSG: polysomnography.

In addition to the three main module categories, an interface is a convenience module built on the Epicurrents application programming interface (API). Using an interface module is optional, but it can make it easier to work with the API and wrap the library into a usable application. Unlike the other module types, only one interface module can be active at a time.

Processing larger amounts of data can be demanding on the central processing unit, and the library uses web workers to handle much of this work in separate processor threads. Although this asynchronous programming style is architecturally more challenging, it benefits the end user, as intensive processing tasks will not result in unresponsiveness (i.e. ‘freezing’) in the application interface. Transferring data to web workers usually requires duplicating it in the receiving processor thread, which could significantly increase the memory requirement of the application. To work around this limitation, Epicurrents can store larger amounts of signal data in shared memory buffers, avoiding the need to duplicate them. However, using shared memory buffers imposes additional requirements on the application’s operating environment, as explained below in section 2.3.

### 2.1 Accessing study and annotation data

Using the web browser as an intermediary, data for a given study can be opened from the local file system or downloaded over an internet connection. Local files can be accessed through the FileSystem API, which is available in many modern browsers, or by utilising the native file input element if the FileSystem API cannot be used. Opening multiple files in the same folder is also supported.

Epicurrents uses the Web-based Distributed Authoring and Versioning (WebDAV; Clemm et al., 2004), a general-purpose web-based file management protocol, to access study data and annotations from remote sources. It is possible to set one or more external WebDAV-compliant data repositories as study and annotation sources. This way, the same study data can be used with different annotations for educational and scientific projects, depending on the source used. Annotation sources can be added in one of three modes (Table 2). A WebDAV server can be set to require user authentication, and it is possible to use different authentication credentials with each WebDAV source. It may also be possible to develop specific access protocols for data providers that do not support WebDAV, but these would have to be examined on a per-case basis.

**Table 2:** Different annotation access modes, what rights they give to the user, and example use case for each mode.

Mode	Access rights	Example use case
Read-only	The user can view annotations in the source but cannot modify them.	Educational examples.
Write-only	The user can save their own annotations in the source but cannot see annotations created by other users.	Scientific annotation projects where different annotators need to be blinded to each other’s answers. Annotation back-up sources.
Read-write	The user can read all annotations and save their own annotations in the source.	General purpose annotation sources.

### 2.2 Epicurrents EEG viewer

To test how the Epicurrents library could be utilised in clinical neurophysiology education and research, we built an EEG viewer application (later referred to as the Epicurrents application) using the following three modules: the EEG study module, the European Data Format (EDF) file reader, and the Python programming language (https://python.org) interpreter service. For general utility, we developed a build for a standalone application that can be installed on the device as a PWA. For a cloud integration demonstration, we developed a plugin for an open-source, Health Insurance Portability and Accountability Act −compliant cloud platform, Nextcloud (https://nextcloud.com). This plugin can be used to open data files from the cloud and examine them in the browser, as well as directly save annotations in the same cloud repository.

The EEG module version used in this trial requires the EDF file reader to read raw signal data. The EDF reader supports both the original EDF file format and the later extended EDF + format. Although much less often used, the BioSemi variant of these formats (BDF and BDF + ) is also supported. Raw signals are matched with electrode names from the International 10–20 system (Jasper, 1958) and can be organised into different montages. Configurations for the recording system and different montages are read from external JavaScript Object Notation files. This gives the ability to extend the existing configuration and add custom montages without needing to modify the application source code.

**Fig. 2:**
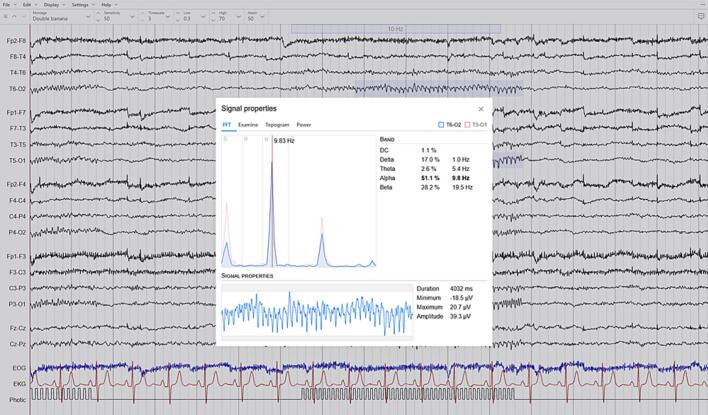
Signal analysis features. Epicurrents provides a number of basic tools for signal analysis. With the Fast Fourier transform tool, frequency properties can be calculated and compared between multiple signal segments (screen capture from the Epicurrents software).

**Fig. 3:**
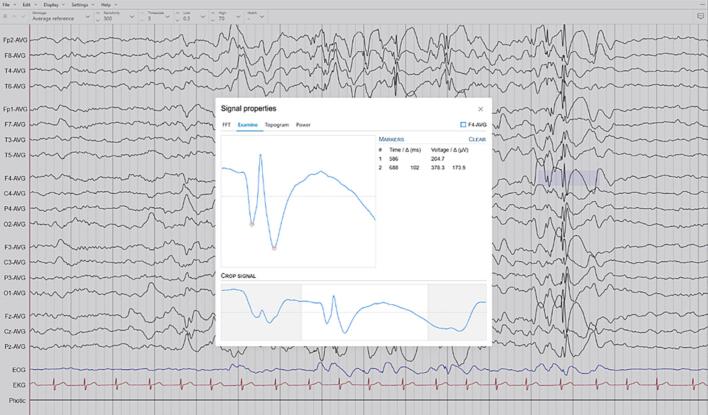
Waveform analysis features. Selected signal segments can be cropped down to individual waveforms for closer examination (screen capture from the Epicurrents software).

EEG-signals organised into montages can be filtered with high-pass and low-pass filters as well as band-reject (e.g. notch) filters using a fast Fourier transform (FFT) algorithm. The same algorithm can also be used to calculate and compare frequency spectrums from individual signal segments (Fig. 2), and these signal segments or the waveforms they contain can be examined more closely (Fig. 3).

**Fig. 4:**
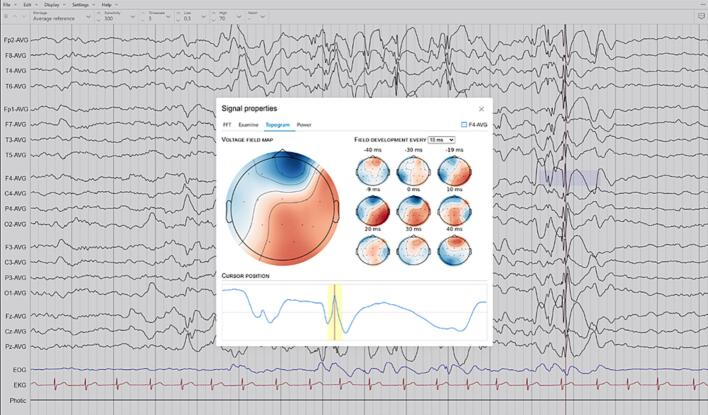
Advanced signal analysis. The MNE Python integration makes it possible to examine voltage maps and field development series for spikes and other waveforms in Epicurrents (screen capture from the Epicurrents software).

The native JavaScript FFT implementation used by the library is relatively lightweight and is usually sufficient for educational use and demonstrations. For scientific use, the EEG module interfaces with the Python interpreter service, which can use most of Python’s scientific libraries for signal processing, including SciPy (https://scipy.org) and MNE (https://mne.tools). In addition to more advanced filtering algorithms, this integration offers access to a host of other signal processing tools, such as voltage field calculation (Fig. 4). However, using the Python interpreter service carries a higher memory requirement and may not be available on all devices.

### 2.3 Limitations and potential weaknesses of JavaScript applications

JavaScript has limited support for memory management. Depending on the active modules, the initial memory requirement for using the library can be considerable, which may rule out mobile devices with less working memory. Opening large recordings for computational analysis may not be possible if the amount of signal data exceeds the memory available to the web browser. Estimated memory requirements for each currently available Epicurrents feature are listed in Table 3.

**Table 3:** Estimates of typical memory requirements per Epicurrents feature.

Feature	Estimated memory requirement
Base library	200 MB
Python interpreter (including SciPy and MNE)	500 MB
ONNX model support	200 MB
Physiological signals in EDF format	2–3 MB per 1 MB of EDF*

* BDF has a slightly lower factor of memory conversion

As a scripting language, JavaScript is limited to running in one processor thread (if not using web workers, detailed below) and cannot reach the general performance of compiled program code, although optimisations in the Chrome V8 JavaScript engine are closing this gap (Momtchev, 2021). Epicurrents depends on WebGL (Web Graphics Library) to display multiple simultaneous signal channels, and additional downsampling may be required to smoothly display high-density data.

As a general security measure, the more advanced memory management features used in the Epicurrents application require the web servers hosting the application to employ a cross-origin isolation policy (Cross-origin isolation, 2025). If this policy is not used, the application features that depend on shared memory buffers will be automatically disabled.

### 2.4 Data security

Data security is of great importance when personal and medical data are concerned. Legislations like the General Data Protection Regulation (Data protection under GDPR, 2025) in the European Union govern access to non-anonymised medical data and other personal information and must be considered when systems processing such data are designed.

Data security can be approached using the confidentiality, integrity, and availability (CIA) triad (Nieles et al., 2017). Since Epicurrents is meant for educational and scientific use only, the data it processes should be deidentified and not contain any personal information. As an added security measure, data from fields that usually contain personal information (such as certain fields in the EDF header) are not displayed to the user. Epicurrents itself does not store any data (availability) and does not contain any authentication features (confidentiality). User authentication must be handled by the data storer, be it the user’s device itself (e.g. an operating system on a desktop computer) or an online data repository (e.g. a cloud service). When accessing data from a remote source, a secure socket layer connection can be used to guarantee that the accessed information is not visible to outsiders. Moreover, both the Epicurrents application and the remote server can be configured to instruct the application user’s browser and all intermediary proxies to not cache the accessed data, which further reduces the risk that a third party could gain access to it.

Identifying and eliminating security vulnerabilities in web applications require vigilance and compliance with security guidelines, such as the Open Web Application Security Project Top 10 (About OWASP, 2025). Some of these security risks can be mitigated with automated code tests and regular software dependency auditing, but data security must always be considered in the primary design of the software as well.

For additional security, both the data and the service hosting the Epicurrents application can have limited access (e.g. within a hospital’s internal network) instead of being accessible through the internet. More in-depth coverage of secure web server configurations is beyond the scope of this article, but such configurations should be easily handled by any professional web server administrator.

## 3 Results

### 3.1 Educational use

The Epicurrents application was used as an EEG case presentation platform for hands-on courses at the 2024 and 2025 congresses of the European Academy of Neurology (EAN). On the platform, course attendants can browse EEG samples featured in the lectures on their personal laptops using a web-based, interactive EEG viewer. The concept has been well-received, and similar courses are planned for future EAN congresses.

### 3.2 Research use

**Fig. 5:**
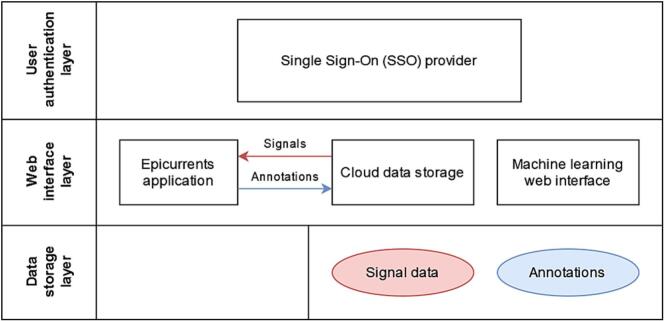
Schematic for a signal data annotation setup for machine learning. This example setup uses the same immutable signal data for creating annotations and ML model training. An Epicurrents application is used to open signal data from the cloud storage provider and save annotations on the same cloud. The machine learning web interface provides access to a model training platform that has direct access to both the signal data and the annotations. As the top layer, an SSO provider manages user authentication and access to different resources.

The Docker container application platform (Docker Hub Container Image Library) can be used to quickly deploy flexible single- and multi-application cloud services. Using Docker and the Epicurrents Nextcloud plugin, we created a convenient, cloud-integrated neurophysiological signal-data review and annotation platform. In this setup, Nextcloud allows for managing the datasets using a familiar, filesystem-like interface and controlling access to the data for each user or group of users. Epicurrents is used to open and display the signal data in a standardised manner and then to save any created annotations to the same cloud repository. JupyterHub, a multi-user Jupyter notebook manager (https://jupyter.org), is used to provide a web interface for designing and training ML (machine learning) models using Python. As both environments can be given access to the same data, no files need to be moved around between annotation and model training. Additionally, trained models can be stored in the same cloud repository in an ONNX format, opened with Epicurrents, and tested using new or existing data through the same application interface. A single sign-on (SSO) provider controls user access to each of the resources, so different users may have access to the annotation environment and the model training environment (Fig. 5).

### 3.3 Machine learning features

Incorporating ML features into Epicurrents requires access to trained models (also known as algorithms) in an ONNX format. To receive meaningful results, these models usually require data to be pre-processed in a certain way before processing with the model itself. The pre-processing pipeline used when training the model may have included advanced signal processing tools or particular program libraries. By using the Epicurrents Python integration, an identical pre-processing pipeline can be achieved compared to the model training scenario if the model was trained in a Python environment. In cases where some other programming language was used, the advanced data processing tools offered by Python scientific libraries may still achieve much closer parity than mere JavaScript.

A research group from Helsinki University Hospital, Finland, released a dataset of neonatal EEG recordings and seizure annotations, as well as a set of ML algorithms for detecting neonatal seizures developed using this dataset (Stevenson et al., 2019). As an example of an ML model integration, this dataset and two of the ML models were used to create an integrated EEG dataset viewer, which is available at https://demo.epicurrents.ai/seznet.

### 3.4 Weaknesses

Due to limitations with memory management in JavaScript, the size of a recording that can be processed with the Epicurrents application depends on the memory available to the web browser. The memory management of the Epicurrents library has been designed to allow going through large numbers of smaller individual recordings. However, opening larger recordings consecutively may exhaust the web browser’s memory if the inbuilt JavaScript garbage collector does not have enough time to free up the memory released by closing previous recordings.

Epicurrents is not a turnkey solution for every scenario. Its flexibility comes with complexity, and using it in a project may require additional setup. Documentation and user guides are an integral part of a project like this and must be maintained alongside the library itself. User documentation is continuously updated and can be accessed through https://docs.epicurrents.io.

### 3.5 Not for clinical diagnostic use

The current version of the Epicurrents library is meant purely for educational and scientific use. It is not a CE-marked medical device software in accordance with the Medical Devices Regulation (Regulation (EU) 2017) and, thus, should not be used for medical diagnostics or clinical decision-making.

## 4 Discussion

Web applications offer considerable advantages when it comes to accessibility, maintainability, and cost compared to traditional desktop software. In the field of radiology and nuclear imaging, web-based applications are gaining popularity, and widely adopted open-source alternatives already exist for scientific use (Ziegler et al., 2020). The combination of accessibility, flexibility, and transparency offered by open-source web application technology allows community-driven design and testing of new ideas, some of which may eventually trickle into everyday workflow.

Following open-source software principles, the source code of Epicurrents is available for anyone to inspect, modify, and distribute (The Open Source Definition and Initiative 2025). Open source provides multiple benefits compared to proprietary software. The most obvious benefit is the cost, as open-source applications are free to use. For projects that employ a continuous integration and deployment strategy, small updates and fixes to the software are applied continuously. As the continuation of an open-source project does not depend on its financial success, even projects targeted at small audiences can remain viable. Furthermore, by using public code repositories (e.g. GitHub), the entire development process can be kept transparent. Disadvantages of using open-source software include a lack of dedicated technical support, often less comprehensive or incomplete documentation, and reliance on an active community to continue developing the software (Androutsellis-Theotokis et al., 2010). In addition to traditional human-generated code, the concept of open source can be extended to machine-generated properties in an AI algorithm (The Open Source Definition 2025), and Epicurrents is designed with support for open AI models in mind. Open source and proprietary can be considered two extremes on a spectrum of approaches, and many projects employ a hybrid strategy, maintaining many of the advantages of open source while still having a degree of financial support for continued development.

Various formats for storing clinical neurophysiology signals have been developed over the years, and many of these formats are proprietary or vendor specific. Out of currently available open data formats, the European Data Format (Kemp et al., 1992, Kemp and Olivan, 2003) and its derivatives (EDF, EDF+, BDF, BDF + ) have become the de facto standard for exporting longer time series data like EEG and polysomnography. Although these formats have proven useful for research purposes, they do not support additional media modalities, like video or audio, and are therefore only partially suitable for data archival. Developing universal standards for the storage of multi-modal (biosignal) data from clinical neurophysiology studies using the Digital Imaging and Communications in Medicine (DICOM) standard is well underway (Halford et al., 2021), but support for the DICOM format in clinical software is still very limited. The Epicurrents project promotes the use of open file formats and supports the EDF/BDF and DICOM formats for EEG recordings, while also providing a framework for adding support for additional file formats.

**Fig. B.1:**
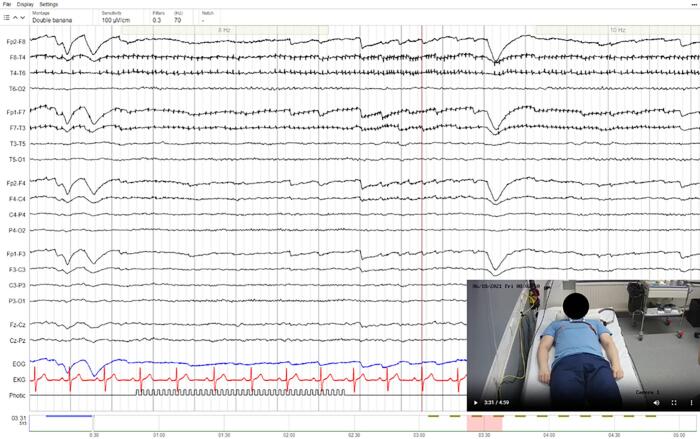
. Screen capture of EEG with synchronised video in the Epicurrents software.

**Fig. B.2:**
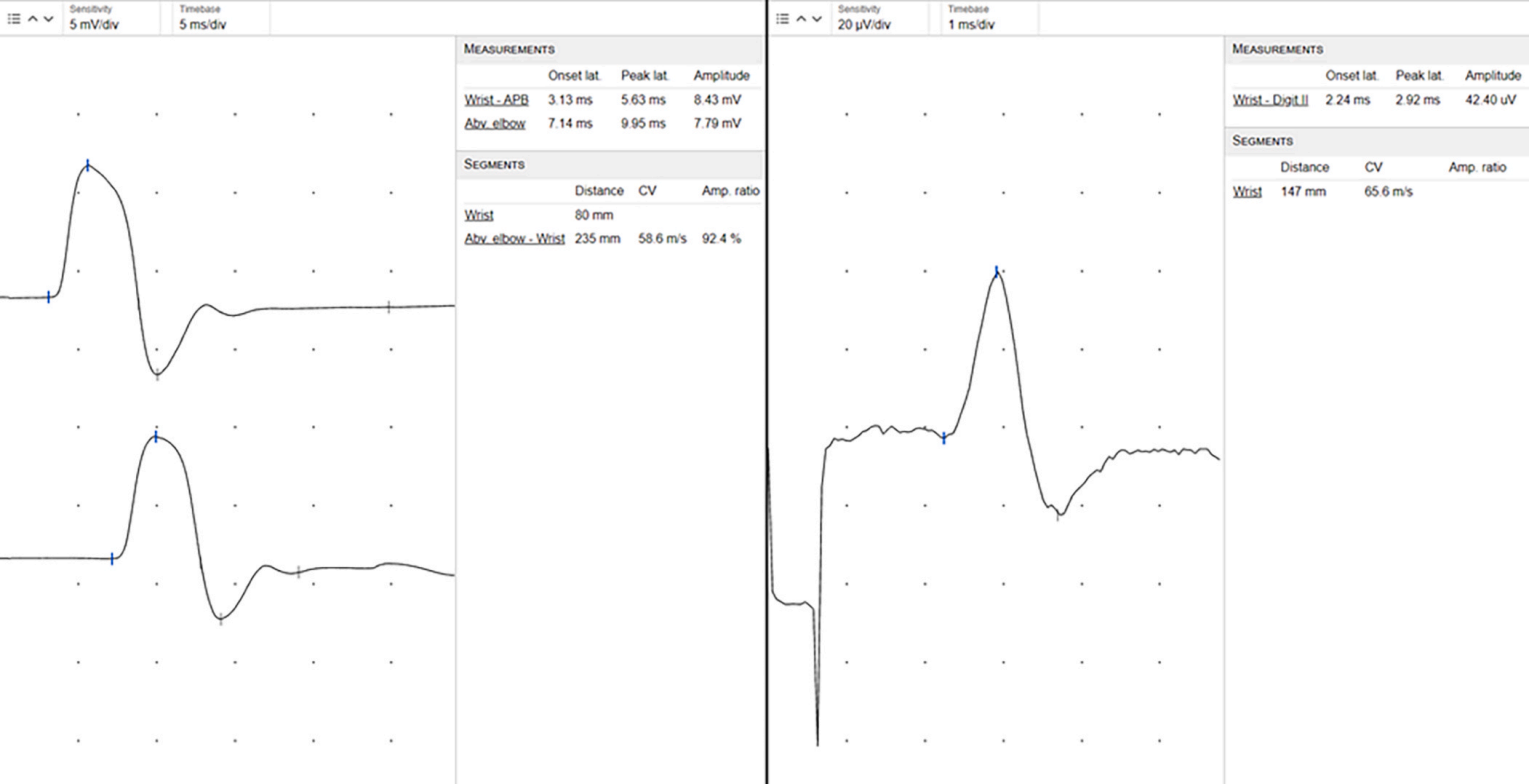
**. Screen capture of median nerve motor and sensory conduction studies in the Epicurrents software**. All the computed values seen in the example figures respond to user interactions, such as moving a latency marker or cursor.

**Fig. B.3:**
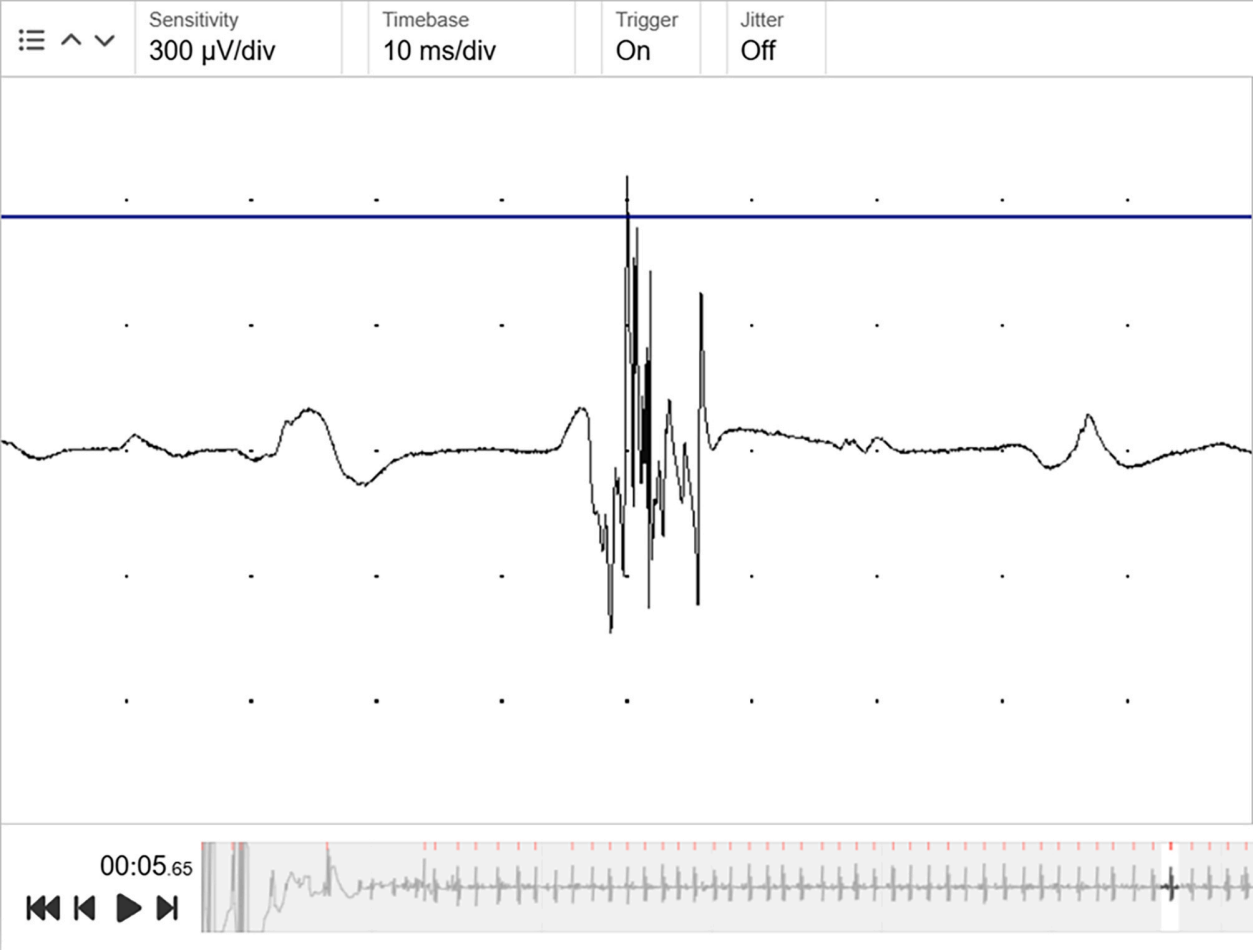
. Screen capture of a triggered electromyography signal displaying a polyphasic muscle action potential in the Epicurrents software.

**Fig. B.4:**
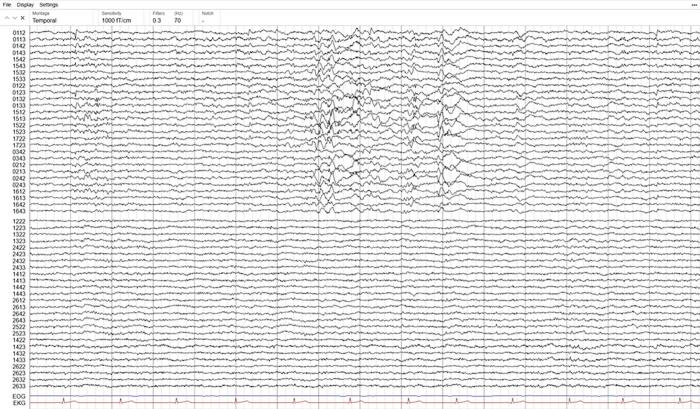
. Screen capture of an MEG recording displayed in a temporal montage in the Epicurrents software.

In addition to plain EEG recordings, the Epicurrents project includes working prototypes of study modules for video EEG (Fig. B.1), NCSs (Fig. B.2), EMG, and triggered single-fibre EMG (Fig. B.3) with accompanying file readers for a few vendor file formats. An adaptation of the EEG study loader can be used to view magnetoencephalography data (MEG; Fig. B.4). All study types support changing the sensitivity and timebase of the display. Additionally, EEG and MEG studies support custom montages and signal filtering. The library itself is not limited to supporting only neurophysiological signal studies and already has modules for simple text-based documents and PDF files. It is technically possible to add support for other medical diagnostic modalities, such as radiological imaging studies. This way, Epicurrents could potentially be used as a multi-modal platform for medical diagnostic education or as a review and annotation interface for large research data repositories.

Since it is an open-source project, anyone can contribute to the development of the Epicurrents library, and contributors do not have to be professionals in the field of medicine or software development. An active community forms the foundation of open-source projects, and projects with large communities can produce software that rivals commercial alternatives in their field, exemplified by the Apache web server (https://apache.org), the Android operating system (https://android.com), and the Chromium project (https://chromium.org). As its development is not driven by financial return, the Epicurrents project has the potential and freedom to grow and evolve to truly meet its users’ needs.

## 5 Future directions

There is an ongoing revolution in many industries thanks to the advancements and heavy investments in AI technologies (Coordinated Plan on Artificial Intelligence, 2025, Announcing The Stargate Project, 2025). Although AI is still an emerging tool in clinical neurophysiology, it is fast becoming a cornerstone in diagnostic medical imaging (Khalifa and Albadawy, 2024). Big tech domination in the consumer AI field is already a concern (Kak, 2023), and commercial operators will no doubt control the development of AI in clinical medicine as well. However, annotating large datasets for ML use is still time consuming, and in the case of EEG, such annotated datasets are only available commercially (Tveit et al., 2023). The signal data annotation platform built with Epicurrents could offer a way to distribute a project’s annotation work to a larger number of people, reducing the cost to each contributor and possibly avoiding the need for massive, centralised funding. This could make it more feasible to annotate datasets of EMG and NCS signals, which have not yet seen the same amount of commercial interest as EEG and polysomnography. Most clinical centres have recorded and collected clinical neurophysiology data in digital format for years now, but many older clinical neurophysiology equipment store their recordings in proprietary file formats where the signal data are not readily available. It is possible to develop file format readers for Epicurrents that can read these files, potentially regaining access to the data recorded by some older devices.

Our next step is to test how Epicurrents can be utilised to convert larger datasets of neurophysiological recordings between open file formats. For this, we will select a set of EEG, NCS, EMG, and polysomnography recordings and use Epicurrents to save them into an openly accessible data repository in extended EDF and limited DICOM formats.

## 6 Conclusion

Epicurrents is an open-source JavaScript library that offers an interactive, state-of-the-art solution for the evolving needs of clinical neurophysiology education. It can be extended to support a range of diagnostic modalities and source file formats. Combined with support for Python code, AI algorithms, and open data standards such as EDF and DICOM, it can also offer a powerful and highly versatile tool for scientific use. As an open-source software library, Epicurrents is not only free to use but is also meant to be reused, modified, and adapted to the needs of its users. Moreover, with the support of an active development community, it can be extended to serve a broad range of educational and scientific needs. Thanks to the constant advancement in web application technology, PWAs like Epicurrents do not appear to be a temporary phenomenon but instead seem to have increasing potential in both non-clinical and clinical use.

## Author contributions

SL designed the trial software and coordinated its use with the collaborating parties. SL, PJ, and EM drafted the manuscript. RK and EM contributed to reviewing the study design. All authors have reviewed the manuscript and approved it for publication.

## Declaration of competing interest

The authors declare that they have no known competing financial interests or personal relationships that could have appeared to influence the work reported in this paper.

## Glossary of technical terms

API: Application Programming Interface; a software component that allows other software to communicate with the application.
BDF: BioSemi Data Format; a variant of the European Data Format using 24 bits per signal sample instead of 16 bits.
Docker: an open-source platform for building containerized applications.
DICOM: Digital Imaging and Communications in Medicine; an open standard used primarily for transferring and archiving medical imaging data.
EDF: European Data Format; an open file format for storing time series signal data.
FFT: Fast Fourier Transform; an efficient algorithm for converting signal data from time domain to frequency domain.
JavaScript: a scripting language for creating dynamic website content and applications that can be run inside a web browser.
JavaScript Object Notation: a text-based format for storing data in key-value pairs, following the JavaScript object structure.
ML: Machine Learning; a subfield of artificial intelligence that uses historical data to develop algorithms (models) for making predictions on new data.
MNE: a Python programming library for human neurophysiological data.
ONNX: Open Neural Network Exchange; an open format for presenting machine learning models in a standardized way.
PWA: Progressive Web Application; a website that has been designed to function similarly to native applications and can be installed on the machine for offline use.
SciPy: a Python programming library for scientific computing (including signal processing).
SSO: Single Sign-On; an authentication method for using a single set of access credentials for multiple websites, applications or services.
WebDAV: Web-based Distributed Authoring and Versioning; an extension of the HTTP protocol for remotely accessing and managing files on a web server.
WebGL: Web Graphics Library; a JavaScript API for rendering high-performance graphics in a web browser.
Zero footprint: a design principle for accessing application over a network and running them entirely in the main memory, leaving no installation files (footprint) on the device.

## Appendix A: Online resources

Project website: https://epicurrents.io.

Project repository: https://github.com/epicurrents.

Development test version: https://alpha.epicurrents.io.

Basic demonstrations: https://demo.epicurrents.io.

AI implementation: https://demo.epicurrents.ai.
